# The Treatment of Congenital Dislocation of the Hip

**Published:** 1899-10-28

**Authors:** 


### THE TREATMENT OF CONGENITAL DISLOCATION OF THE HIP.

Dr. Royal Whitman,[1] of New York, strongly recommends the open operation for congenital dislocation of the hip, a condition which is to be regarded as a serious and progressive deformity, and one full worthy of careful operative treatment. He thinks that the statements in recent text-books and the discussions at societies show that the gravity of the disability is not appreciated, and that the dangers of positive treatment are exaggerated. The operation which he performs is slightly modified from that of Lorenz, and is done as follows: The joint is opened by a lateral incision just below and one inch to the outer side of the anterior superior spine. This exposes the fascia, through which the line of junction between the tensor vaginæ femoris and the gluteus medius muscles may be seen. When these are separated from one another the capsule of the joint is at once exposed, often covered in parts by the fibres of the ilio-psoas muscle. The capsule is opened by an incision in the line of the neck of the femur, and through this the head of the bone may be exposed after the division of the ligamentum teres, if it be present. The finger may then be passed through the opening downward and forward to the acetabulum, and by its side a strong cervix dilator is inserted, with which the capsule is thoroughly stretched especially the anterior layer which covers the acetabulum. The acetabulum is excavated to the extent that seems desirable by means of a sharp spoon, after which the head of the bone is replaced. The wound is closed, except for a small gauze drain enclosed in rubber tissue, and a plaster-of-Paris spica bandage, including the foot, is applied, with the leg in an attitude of moderate abduction, complete extension, and slight inward rotation. In older patients a more extensive division of the capsule may be required.

